# Brachytherapy in craniopharyngiomas: a systematic review and meta-analysis of long-term follow-up

**DOI:** 10.1186/s12885-024-12397-1

**Published:** 2024-05-24

**Authors:** Li-Yuan Zhang, Wei Guo, Han-Ze Du, Hui Pan, Yun-Chuan Sun, Hui-Juan Zhu, Shuai-Hua Song, Xiao-Yuan Guo, Yue Jiang, Qian-Qian Sun

**Affiliations:** 1 Department of Endocrinology, Key Laboratory of Endocrinology of National Health Commission, State Key Laboratory of Complex Severe and Rare Diseases, Peking Union Medical College Hospital, Chinese Academy of Medical Science and Peking Union Medical College, Beijing, 100730 China; 2 Department of Radiation Oncology, Hebei Province Cangzhou Hospital of Integrated Traditional and Western Medicine, Cangzhou Hebei, Hebei 061000 China; 3 Department of Endocrinology, The ninth Hospital of Xingtai, Xingtai, Hebei 055250 China

**Keywords:** Brachytherapy, Craniopharyngiomas, Irradiation, Efficacy

## Abstract

**Objective:**

Brachytherapy has been indicated as an alternative option for treating cystic craniopharyngiomas (CPs). The potential benefits of brachytherapy for CPs have not yet been clarified. The purpose of this work was to conduct a meta-analysis to analyze the long-term efficacy and adverse reactions profile of brachytherapy for CPs.

**Materials and methods:**

The relevant databases were searched to collect the clinical trials on brachytherapy in patients with CPs. Included studies were limited to publications in full manuscript form with at least 5-year median follow-up, and adequate reporting of treatment outcomes and adverse reactions data. Stata 12.0 was used for data analysis.

**Results:**

According to the inclusion and exclusion criteria, a total of 6 clinical trials involving 266 patients with CPs were included in this meta-analysis. The minimum average follow-up was 5 years. The results of the meta-analysis showed that 1-year, 2–3 years and 5 years progression free survival rates (PFS) are 75% (95%CI: 66-84%), 62% (95%CI: 52-72%) and 57% (95%CI: 22-92%), respectively. At the last follow-up, less than 16% of patients with visual outcomes worser than baseline in all included studies. While, for endocrine outcomes, less than 32% of patients worser than baseline level.

**Conclusion:**

In general, based on the above results, brachytherapy should be considered as a good choice for the treatment of CP.

**Supplementary Information:**

The online version contains supplementary material available at 10.1186/s12885-024-12397-1.

## Introduction

Craniopharyngiomas, often abbreviated as CPs, are classified as benign neoplasms with an epithelial character, typically emerging from two primary origins: remnants of the craniopharyngeal duct from embryonic development, known as the adamantinomatous variety, or as an unusual cell growth phenomenon within the adenohypophyseal tissues, identified as the squamous papillary variant [1, 2]. These tumors remain relatively infrequent worldwide, presenting an incidence rate approximated at 0.13 occurrences per 100,000 individuals annually [3]. CPs has bimodal distribution across age groups, with significant prevalence in young individuals and a subsequent resurgence in adults aged 50 to 74 years. Such a pattern underscores their prominence as the leading type of nonglial brain neoplasm among pediatric cohorts, contributing to between 5.6% and 15% of all pediatric central nervous system malignancies [3, 4]. A characteristic feature of CPs involves the presence of a cystic structure in an estimated 90% of instances [5, 6], prompting the categorization into distinctive radiological classifications: isolated cystic formations, predominantly solid masses, or a composite of cystic and solid structures [7].

The recognized optimal approach for managing CPs hinges on executing the most extensive yet safe surgical extraction possible, which is often succeeded by administering radiotherapy if residual tumor tissue persists [8, 9]. Nonetheless, a contemporary shift in strategy has emerged, particularly concerning pediatric patients. This change advocates for postponing more invasive treatment protocols in younger populations. The rationale behind this more cautious approach stems from the heightened susceptibility of pediatric patients to an array of severe complications. Specifically, this demographic demonstrates increased vulnerability to visual impairments, disruptions in endocrine function, and cognitive deficits following aggressive treatment measures [10–14].

For CPs that exhibit cystic elements, various therapeutic interventions are available. Strategies employed are the administration of substances directly into the cyst, including bleomycin, alpha interferon, and specific radioisotopes [15–17]. It is crucial to recognize the potential risks associated with these interventions. For instance, unintended leakage of bleomycin can lead to adverse neurotoxic effects [15, 16]. In addition, selected research has showcased promising outcomes following procedures such as cystic fenestration [18, 19].

Radiation therapy for benign diseases is not a new method and has become a useful treatment modality since the discovery of radiation more than a century ago [20]. Brachytherapy has been acknowledged as a therapeutic alternative for cystic CPs, tracing back to its initial documentation seven decades prior. Successive publications by various scholars have presented their patient groups, underscoring the effectiveness of this treatment modality while maintaining complication rates within acceptable parameters. The methodology involving the instillation of radioisotopes serves the purpose of achieving tumor management, favoring a less invasive approach that circumvents the need for patient exposure to external radiation sources [16, 21, 22].

In the span of recent years, advancements have been notable in the realm of brachytherapy techniques, accompanied by enhancements in the trials exploring this subject, collectively contributing to the heightened safety parameters of this treatment modality [21, 22]. In addition, emerging insights in the field of immunohistochemistry, coupled with a deeper understanding of the morphological attributes of CPs and their responsiveness to brachytherapy, are poised to forge a novel path of comprehension in this domain [23].

At present, the scarcity of brachytherapy facilities can be attributed to the necessity for sophisticated nuclear infrastructure, a factor that significantly limits their ubiquity [16, 24]. Moreover, the duration of follow-up in pertinent studies remains insufficiently extensive, giving rise to a dearth of uniform, contemporary data that attest to the efficacy and potential morbidities associated with this therapeutic approach. This gap in the literature underscores the imperative for the execution of this current systematic review and meta-analysis, designed to collate and scrutinize the available evidence in a comprehensive manner.

## Materials and methods

### Literature search

This meta-analysis adhered strictly to the criteria set forth by the Preferred Reporting Items for Systematic Reviews and Meta-Analyses (PRISMA) [25]. Ethical approval was not necessary as the study did not involve individual patient data. A thorough literature exploration was conducted from January 2010 to August 2023, encompassing extensive database searches that included PubMed, Embase, and Web of Science. The following search terms or keywords were used: “craniopharyngioma” OR “cystic craniopharyngioma” AND “intracavitary irradiation” OR “intracystic irradiation” OR “brachytherapy” OR “radioactive” OR “radioisotopes”.

### Study selection

Inclusion criteria mandated for the studies’ selection were meticulous and multifaceted: (a) histological confirmation of CPs in all patient cases, (b) explicit criteria for case selection within the study, (c) a minimum follow-up duration extending beyond five years, (d) availability in complete text format, and (e) publication in English language. Conversely, grounds for the exclusion of studies encompassed: (a) the presentation of data deemed insufficient, (b) follow-up periods considered inadequate, (c) studies primarily based on animal testing, (d) content forms such as letters, abstracts from conferences, or literature reviews, and (e) non-availability in the English language.

### Quality assessment of publications

Evaluation of non-randomized controlled trials was conducted utilizing the Methodological Index for Non-Randomized Studies (MINORS). This index assesses various elements, including the explicit articulation of the research objective, the consistency in patient enrollment, and the prospective nature of data accumulation. A superior score is indicative of enhanced study integrity and methodological rigor.

### Data extraction

Data extraction from the qualified studies followed a structured protocol, undertaken by two investigators (one endocrinologist and one radiation oncologist). Details encompassing the primary author’s identity, publication year, research design, follow-up period, number of participants, demographic details (such as age), cystic volume parameters, specific radioisotopes utilized, administered radiation dosages, therapeutic measures preceding brachytherapy, subsequent treatment modalities, and associated adverse events were meticulously cataloged. In instances of discrepancies or interpretational differences, one senior radiation oncologist provided an additional layer of scrutiny, facilitating a consensus-driven resolution process.

### Statistical analysis

Clinical outcomes, where accessible, were directly retrieved from individual studies or inferred from unprocessed data, employing methodologies delineated by Tierney et al. [26]. Evaluation of result consistency across combined data utilized Cochran’s Q test coupled with the Higgins I² statistic. Implementation of the random-effects model ensued when the I² value exceeded 50%, complemented by a P-value below 0.1, indicative of pronounced heterogeneity. Conversely, conditions not meeting these thresholds warranted the application of a fixed-effects model. Supplementary to these methods, sensitivity analyses meticulously examined the influence of distinct studies on the comprehensive estimation. The potential for publication bias underwent assessment through Begg’s funnel plot inspection. All statistical computations received facilitation through STATA 12.0 software (Stata Corp, College Station, TX, USA). The demarcation for statistical significance stood firmly at a P-value not exceeding 0.05.

## Results

### Literature search and summary of studies

**Fig. 1:**
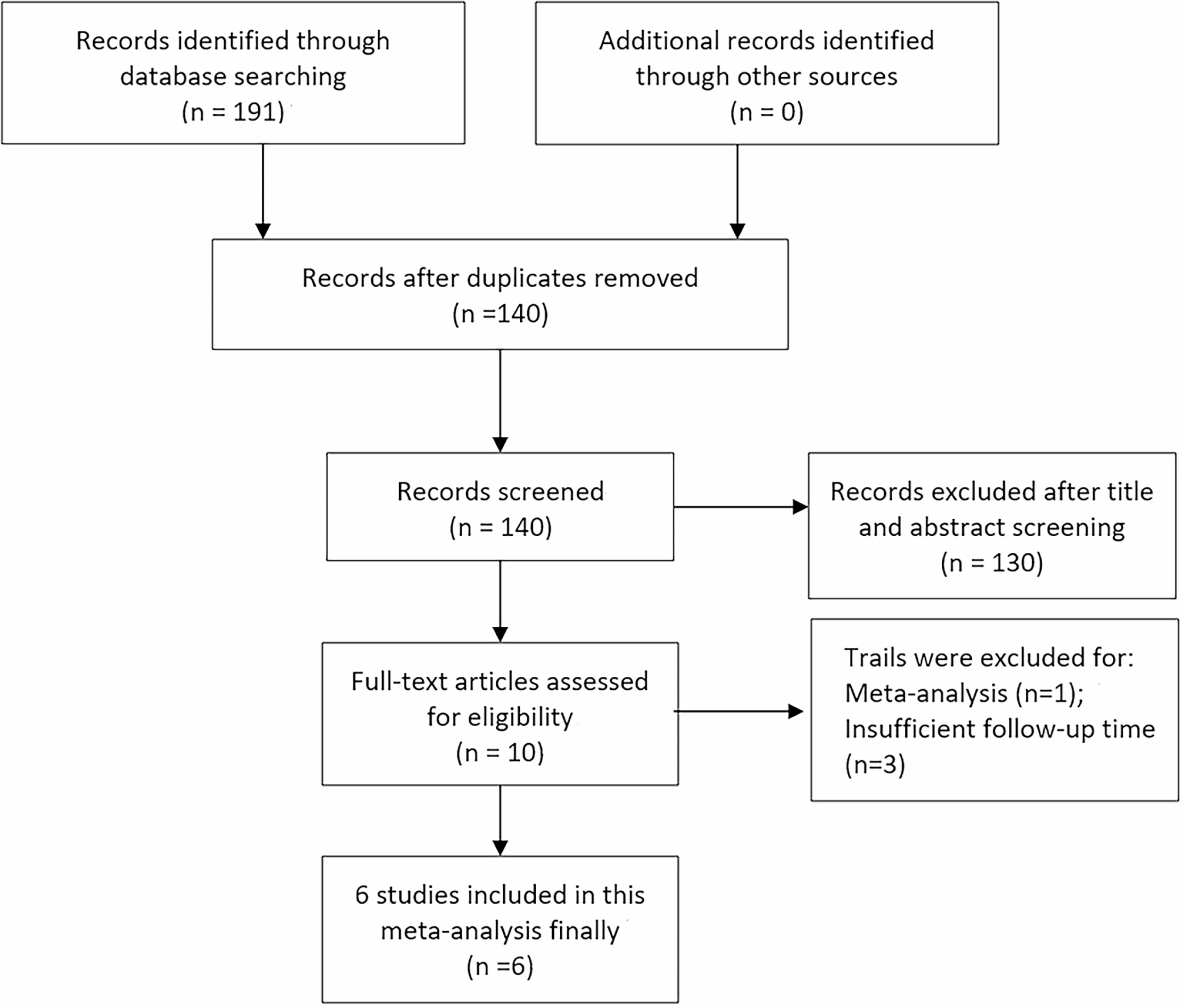
Flow chart of the included trials

The initial search strategy identified 191 potentially relevant studies. Removal of redundant publications left 140 articles for further consideration. An in-depth evaluation of titles and abstracts led to the exclusion of 130 studies, primarily due to irrelevance or insufficient data. Subsequent rigorous scrutiny of the full-text articles and their data quality necessitated the dismissal of another four studies, citing various reasons including lack of comprehensive data. Ultimately, six studies met the stringent inclusion criteria, warranting their incorporation into the final meta-analysis [24, 27–31]. The flowchart in Fig. 1 provides a detailed overview of the meticulous article selection process. The methodological robustness of the non-randomized controlled trials underwent assessment via the MINORS criteria, with each study achieving a score of no less than 10, denoting high quality.

A total of 266 patients were encompassed in this meta-analysis, with individual studies contributing between 9 and 90 patients. Table 1 outlines the fundamental characteristics of the studies incorporated. Regarding the nature of the studies, each one employed a retrospective design. The ages of patients at the median point varied, starting from 6.6 years and extending up to 36.6 years. Brachytherapy involved a meticulously calculated radiation dosage, aiming for 200–300 Gy at the cyst walls. Follow-up durations for these patients also differed, with the median times spanning from 60.2 months to as extensive as 432 months. It was common practice for all patients to undergo alternative treatment modalities, encompassing various surgical interventions, both prior to and succeeding brachytherapy sessions.

**Table 1:** Study characteristics

Study	Reference	Year	Study design	Median/mean follow-up (m)	*n* (male/female)	Median/mean age in year (range)	Median/mean cyst volume in ml (range)	Radioisotope	Radiation dosage (Gy)	Treatment prior to brachytherapy (%)	Further therapies (%)	Related complications (%)
Barriger RB	27	2011	Retro.	62 (8–136)	19(14/5)	11 (3–54)	9 (2–58)	32P	300	No treatment (11)	Repeated brachytherapy (16)	Transient abducent palsy (5)
										Resection surgery (73.6)	Resection surgery (21)	
										Cyst aspiration (36.8)	Cystic aspiration/shunt (42)	
										EBR (5)	EBR (26)	
										Two alpha-interferon (10.5)		
Julow JV	24	2013	Retro.	432	78 (40/38)	28 (2.9–73)	15.6 (0.9–110)	90Y	270	No treatment (4)	Repeated brachytherapy (43)	Oculomotor neuropathy (6.4)
										Resection surgery (96)	Resection surgery (23)	Meningoventriculitis (1.3)
											Cystic aspiration (14)	Hypothalamic/thalamic minor vessels damage (2.5)
											Radiosurgery (3.8)	
Kickingereder P	28	2012	Retro.	60.2 (3–196.7)	53 (18/35)	31.1 (7.6–75.7)	6.1 (0.5–78.9)	32P	200	No treatment (26)	Repeated brachytherapy (7.5)	Hemiparesis (2)
										Resection surgery (52.8)	Resection surgery (15)	Septic meningitis (2)
										Cyst aspiration/shunt (47.2)	Cystic aspiration (36)	Transient deterioration (11)
										EBR (13.2)	Radiosurgery (2)	Oculomotor neuropathy (2)
										Radiosurgery (3.8)	Bleomycin (2)	
										Bleomycin (1.9)		
Ansari SF	29	2015	Retro.	78 (12–144)	9(6/3)	6.6 (3–15)	NR	32P	300	Resection surgery (100)	Cystic fenestration/shunt (55)	Hyponatraemia, seizure (11)
											EBR (77.7)	
Maarouf M	30	2016	Retro.	61.9 (16.9–196.6)	17 (9/8)	15.4 (7.6–18.8)	11.1 (0.5–78.9)	32P	200	No treatment (35)	Repeated brachytherapy (6)	NR
										Resection surgery (59)	Resection surgery (18)	
										Cyst aspiration (35)	Cystic aspiration (12)	
										Radiosurgery (6)		
										EBR (12)		
Yu X	31	2021	Retro.	121 (12–144)	90 (51/39)	36.6 (5–66)	21.4 (1.0–55.0)	32P	250	Resection surgery (61.1)	Repeated brachytherapy (9)	Worsening visual acuity (4.4)
										Ventriculoperitoneal shunt (18.9)	Ventriculoperitoneal shunt (3.3)	Third nerve palsy (2.2)
										SRS (6.7)	Gamma Knife radiation therapy (3.3)	Carotid artery occlusion (1.1)
										EBR (8.9)	Endoscopic third ventriculostomy (1.1)	

EBR: external beam radiotherapy; Retro.: Retrospective study; ^32^P: Phosphorous-32, ^90^Y: Yttrium-90; SRS: Stereotactic radiosurgery; NR: Not reported

### Facilities and installations

An optimal radioisotope for therapeutic use is identified as one that exclusively emits beta radiation of elevated energy and possesses the briefest possible half-life. Agents frequently employed for beta emission include phosphorous-32 (^32^P) and yttrium-90 (^92^Y). The characteristics of these agents were summarized in Supplementary Table  S1. The administration of radioisotopes can be conducted via a reservoir, such as an Ommaya catheter, or by the introduction of a stereotactic needle. Utilizing the Ommaya catheter allows for periodic decompression of the cyst, whereas employing a stereotactic needle offers the precision required for addressing multicystic tumors [16]. In multicystic CPs, those having 3–4 cysts with volumes of 1.5–2.5 ml or more, it is recommended to have at least one instillation and/or a maximum two instillations. In cases with potential recurrence, the second instillation is recommended within 2–3 months. Detailed in Table 1, each study delineates the specific radiation dosages, meticulously calculated to permeate the cyst walls during each application.

### Efficacy outcomes

**Fig. 2:**
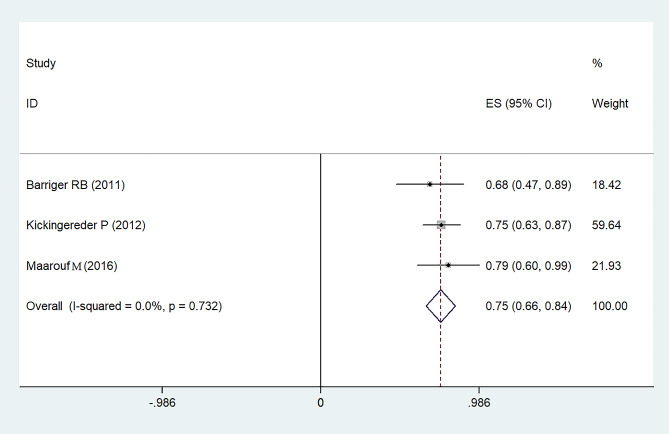
Forest plot for overall 1-year progression free survival rates

**Fig. 3:**
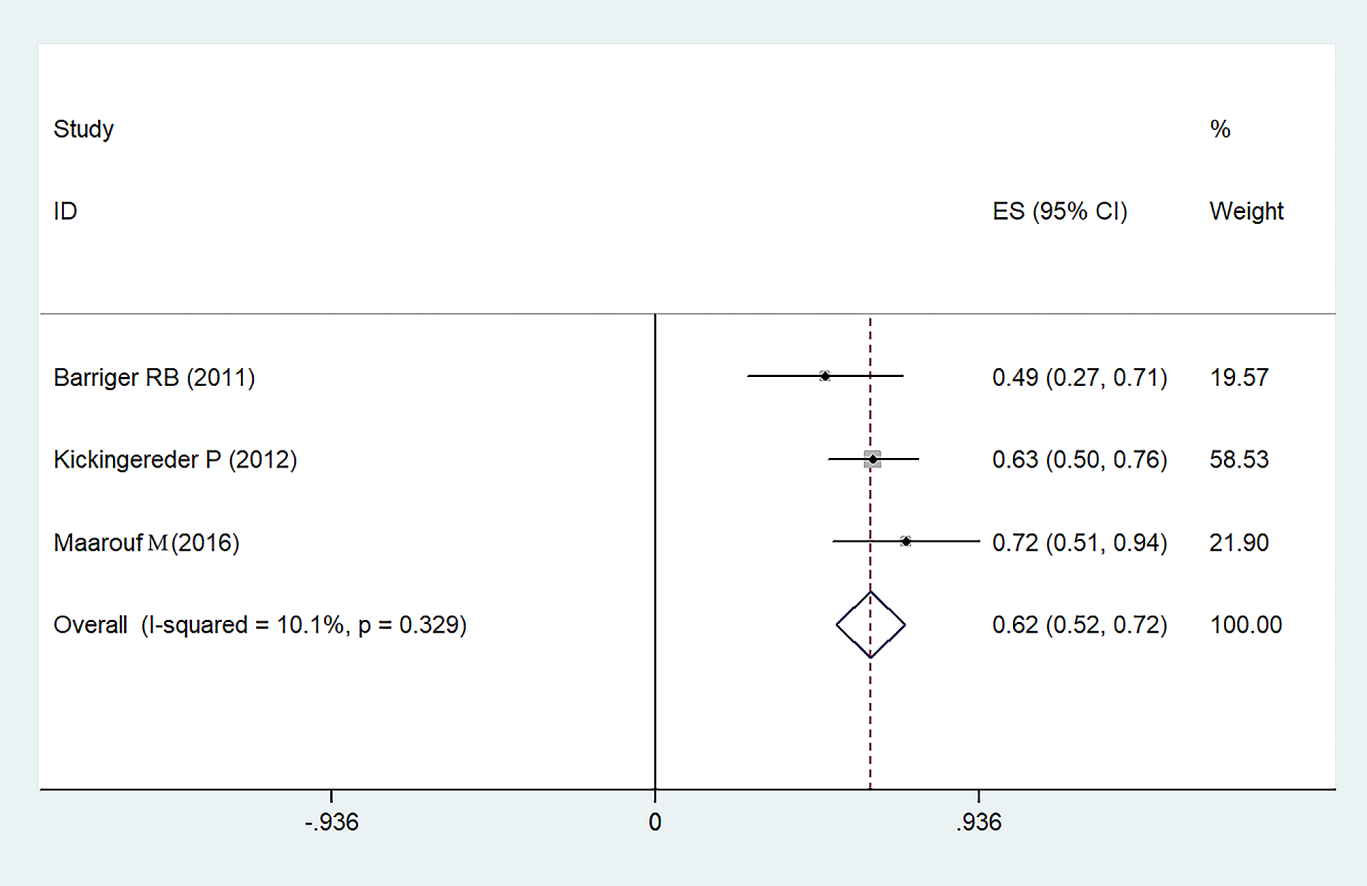
Forest plot for overall 2–3 years progression free survival rates

**Fig. 4:**
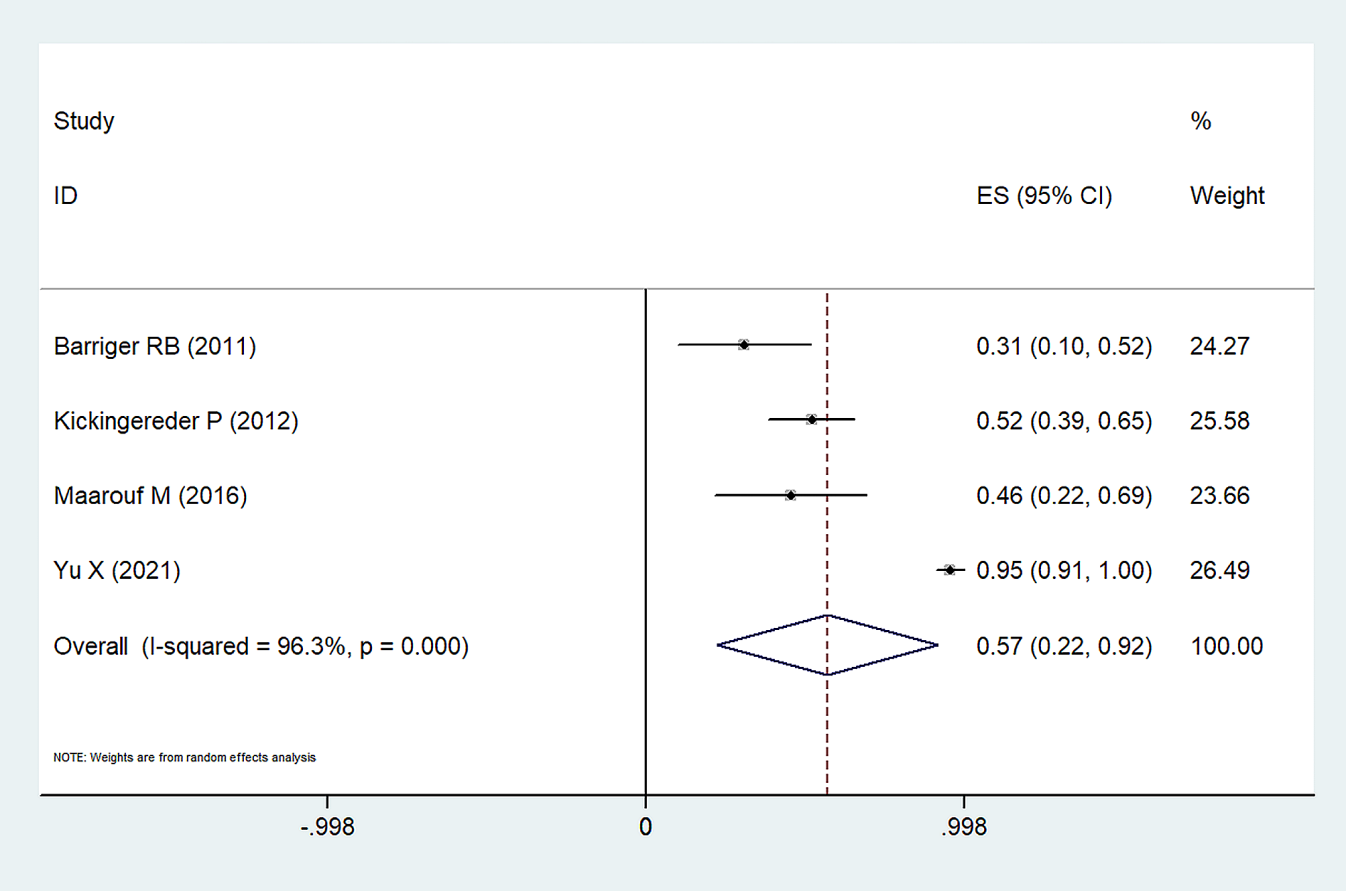
Forest plot for overall 5-year progression free survival rates

Tumor responses, coupled with overall survival (OS) rates, are delineated in Tables 2 and 3. Indications reveal that the 1-year progression-free survival (PFS) rate stands at 75% with a 95% confidence interval (CI) of 66-84% (Fig. 2). Concurrently, the PFS rates for the subsequent 2–3 years and 5 years are documented at 62% (95%CI: 52-72%) and 57% (95%CI: 22-92%), respectively, as detailed in Figs. 3 and 4. A comprehensive representation of the PFS percentages derived from the samples is available in Table 4.

**Table 2:** Tumor response

Study	Complete tumour reduction (%)	Partial tumour reduction (%)	Tumour progression (%)	
Barriger RB	5.2	26.3	47.3	
Julow JV	20.5	56.4	2.5	
Kickingereder P	29.4	52.9	11.8	
Ansari SF	0	22.2	77.7	
Maarouf M	35	29	30	
Yu X	43.4	36.4	16.3	

**Table 3:** Overall survival

Study	OS 5-year (%)	OS 10-year (%)	OS 15-year (%)	OS 20-year (%)	OS 25-year (%)	OS 30-year (%)
Barriger RB	–	–	–	–	–	–
Julow JV	56	29	15	8	3	1
Kickingereder P	–	–	–	–	–	–
Ansari SF	–	–	–	–	–	–
Maarouf M	–	–	–	–	–	–
Yu X	96.7	90	85.5	–	–	–

**Table 4:** Progression free survival

Study	1-year PFS (%)	2–3 years PFS (%)	5 years PFS (%)
Barriger RB	68*/95	49*/95	31*/86
Kickingereder P	75	63	52
Maarouf M	79.4	72.4	45.6
Yu X	100	100	95.5

*Before salvage therapy

### Visual outcomes

The majority of trials analyzed, as summarized in Table 5, indicated either stability or enhancement in visual parameters. Barriger et al. highlighted the most detrimental visual outcomes, primarily attributing them to salvage therapy rather than the effects of intracystic irradiation [27]. Kickingereder et al. noted a pre-therapy visual decline in 66% of participants. However, by the conclusive clinical follow-up, visual enhancement was observed in 25% of these individuals. Instances of diminished visual fields and acuity were evident in 9.6% of participants, although these were not associated with the administration of phosphorus [28]. In a stark contrast, 5.1% of the cohorts studied by Julow exhibited a visual deficit directly attributable to brachytherapy [24]. Yu et al. found that at the final follow-up, a mere 6.1% of participants experienced a deterioration in visual capabilities compared to baseline data [31]. The subgroup analysis revealed that those who had received external beam radiotherapy or stereotactic radiosurgery prior to brachytherapy had a higher severity of visual impairment compared to those who did not receive these treatments (*p* < 0.001).

**Table 5:** Visual outcomes

Study	Unchanged (%)	Worsened (%)	Improved (%)	Uninformed (%)
Barriger RB	52.6	15.8	15.8	15.8
Julow JV	66.7	5.1	26.9	1.3
Kickingereder P	65.4	9.6	25	0
Ansari SF	44.4	0	55.6	0
Maarouf M	76.5	0	23.5	0
Yu X	22.3	6.1	71.6	0

### Endocrine outcomes

Following brachytherapy, Barriger et al. identified a 74% rate of stability within endocrine function, a figure that experienced a decline subsequent to additional salvage therapies. A notable endocrine deterioration was documented in 31.6% of the patient population [27]. In a comparable narrative, Kickingereder et al. observed pre-existing endocrine dysfunction in a predominant segment of patients, with only a singular case demonstrating improvement. Notably, one instance of aggravated endocrine condition was linked to irradiation. Other cases of deterioration were correlated with surgical interventions or the advancement of the tumour [28]. Ansari et al. disclosed a 55.5% incidence of diabetes insipidus, coupled with a surge in hypopituitarism cases by five following surgical procedures, without elaborating on the connection to brachytherapy [29]. Conversely, Maarouf et al. found that out of 17 patients, 16 had pre-existing endocrine dysfunction. In addition, there was the onset of new pituitary deficits in four individuals, independent of disease advancement, potentially implicating brachytherapy [30]. Yu et al., in their conclusive observations, noted that merely 4.9% of participants manifested a more severe endocrine status compared to initial baseline assessments [31]. Comprehensive outcomes are elucidated in Table 6. The subgroup analysis also found that those who had received external beam radiotherapy or stereotactic radiosurgery prior to brachytherapy had a more serious of endocrine impairment compared to those who did not receive these treatments (*p* = 0.001).

**Table 6:** Endocrine outcomes

Study	Unchanged (%)	Worsened (%)	Improved (%)	Uninformed (%)
Barriger RB	68.4	31.6	0	0
Julow JV	–	–	–	100
Kickingereder P	76.9	19.2	1.9	1.9
Ansari SF	88.9	11.1	0	0
Maarouf M	76.5	23.5	0	0
Yu X	32.3	4.9	62.8	0

### Treatment-related complications

Isotope leakage into the subarachnoid space may be an important sequela of radioisotope treatment that injures tissues adjacent to the tumor, such as the optic nerve, hypothalamus, pituitary gland, and internal carotid artery. Neurological toxicities primarily include transient abducent palsy, third nerve palsy and oculomotor neuropathy. The incidence rate ranges from 2 to 6.4%. Vascular injuries primarily include carotid artery occlusion and hypothalamic/thalamic minor vessels damage. The incidence rate ranges from 1.1 to 2.5%. The incidence of encephalitis is less than 2%. The above research findings are presented in Table 1.

### Sensitivity analysis and publication bias

**Fig. 5:**
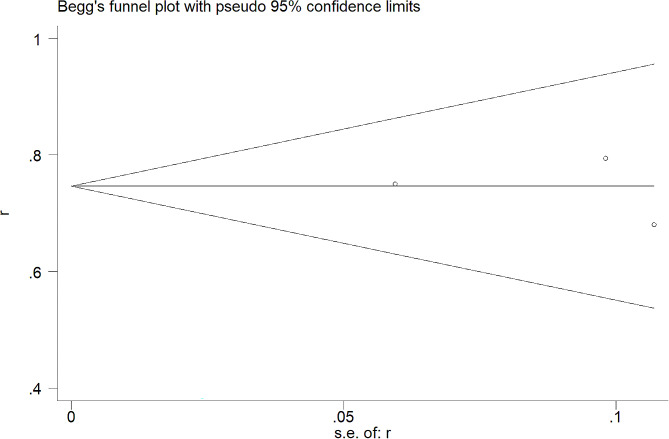
Funnel plots evaluating 1-year progression free survival rates

**Fig. 6:**
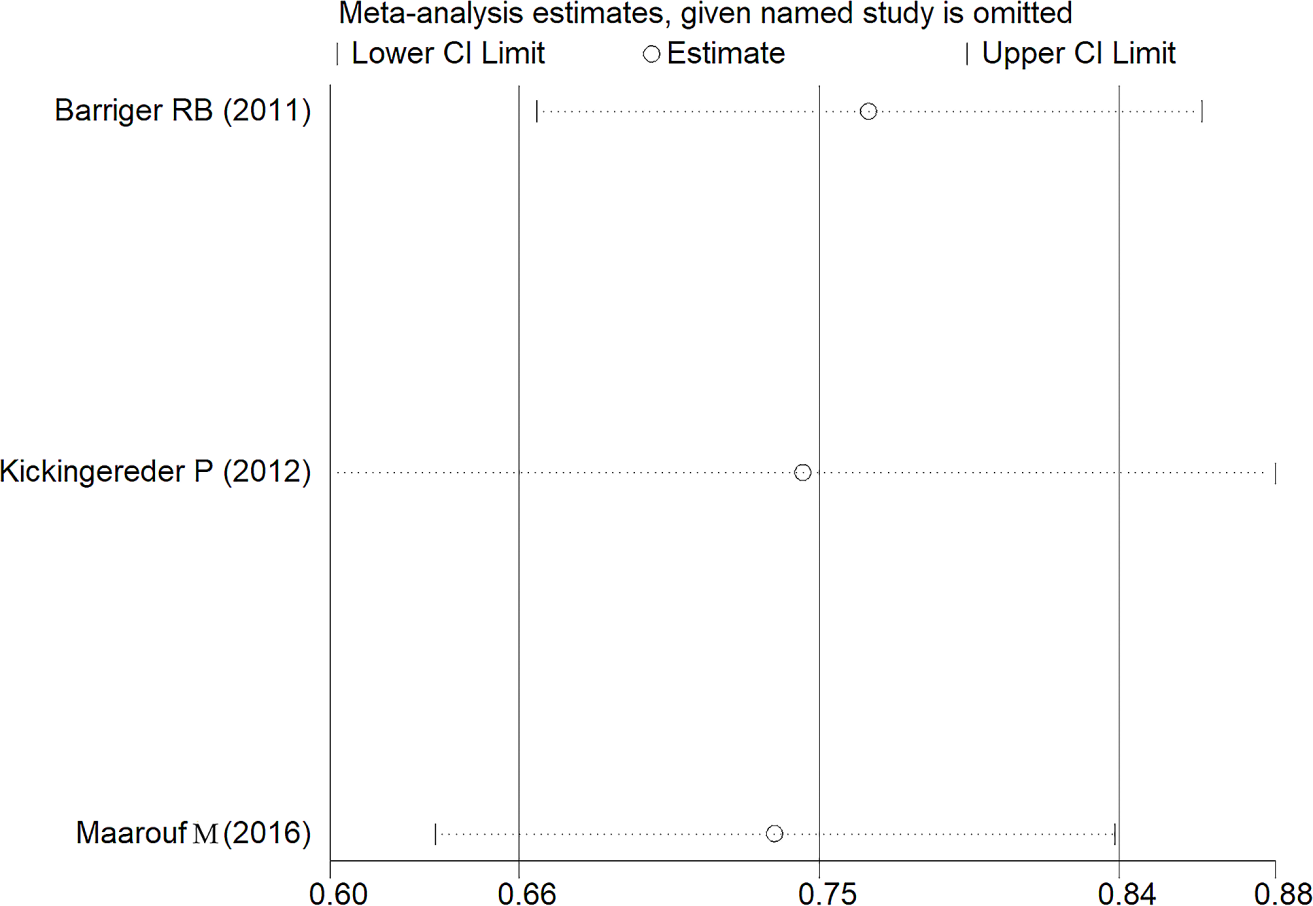
Sensitivity analysis of 1-year progression free survival rates

In the comprehensive meta-analysis encompassing all variables, an examination of the Begg’s funnel plot revealed no substantial asymmetry, as illustrated in Fig. 5. To ascertain the durability of the results from the meta-analysis, a meticulous sensitivity analysis was executed. This rigorous assessment underscored the statistical resilience of the results, confirming that the aggregated outcomes remained unaffected by any single study, as depicted in Fig. 6.

## Discussion

Given the benign nature of CPs, the optimal therapeutic strategy hinges on their full surgical excision [12]. However, achieving this therapeutic benchmark poses significant challenges, particularly while confronting lesions with a predominantly cystic character. The intricate adherence of cystic walls to critical neuroanatomical structures often precludes a comprehensive surgical approach, given the potential for postoperative morbidity surpassing the complications intrinsic to the pathology itself [27]. In scenarios characterizing cystic manifestations, an array of treatment modalities is available, strategically designed to forestall the need for more invasive interventions. These include brachytherapy techniques, the administration of intracystic chemotherapeutic agents, and fenestration procedures, optionally accompanied by aspiration of cystic components, all aimed at mitigating the disease’s progression and attendant symptomatology [14–19].

The inaugural conceptualization of surgical fenestration, involving the cystic walls and their ensuing communication with the ventricular system, was put forth by Cushing in the year 1930 [18, 19]. This methodology demonstrated prompt diminution of the cyst’s volume and adept management of symptoms attributable to compression, albeit with a propensity for heightened recurrence, a trend persistent even amidst adjunctive radiotherapy protocols [9]. Diverging from these outcomes, a study emanating from Egypt documented an unanticipated aftermath following the employment of an Ommaya reservoir, which was positioned utilizing stereotactic puncture for the aspiration of neoplastic fluid in a cohort of 52 cases, primarily composed of cystic CPs. A longitudinal scrutiny extending over seven years revealed a striking 78% of instances evading the necessity for re-intervention [18]. Echoing the insights propounded by Spaziante in parallel case series, the inference shows that the negligible rate of recurrence could be ascribed to the cyst’s conduit with the ventricular framework via the catheter endowed with multiple fenestrations [18, 19]. Contemporary consensus, however, categorizes this modality as palliative, correlated with an escalated incidence of tumoral recurrence, notwithstanding an absence of disparity in the repercussion on protracted health parameters [32].

In the realm of intracystic chemotherapy, the administration of bleomycin has been associated with cystic regulation in approximately 14–71% of instances. Nonetheless, this approach necessitates an assured integrity of the catheter seal, given the potential of the agent to induce grave neurological toxicity. Owing to these perils, utilization of this method has significantly diminished [15–17]. Alternatively, the infiltration of interferon-alpha as an intracystic chemotherapeutic regimen has garnered attention due to its auspicious outcomes noted across various literary works. Instances of cystic regulation have been documented to fluctuate between 22 and 78%, generally accompanied by a minimal spectrum of adverse effects [15, 17]. However, notwithstanding the acceptable indices of neoplastic regulation, the requisite for recurrent, sequential infiltrations marks a downside, potentially culminating in suboptimal adherence to the treatment protocol. Compounding these challenges is the limitation imposed by the treatment’s specificity for a singular cystic entity, a significant concern particularly pertinent in pediatric subjects who exhibit a greater prevalence of multicystic lesions relative to their adult counterparts [15–17, 32].

Brachytherapy, a therapeutic practice with a history spanning seven decades, has consistently yielded outcomes deemed satisfactory [33]. A pivotal study in 1992 by Van den Berge revealed a pronounced reduction in cystic formations across the majority of subjects, albeit accompanied by an escalated incidence of visual impairment, the most pronounced within the cohorts studied [34]. Subsequent research by Pollock identified an 88% reduction rate in cystic structures; however, there was a notable advancement in solid components [35]. Voges’ research delineated complete neoplastic regression in nearly half of the cases examined, and a partial response in one-third of the participants, with complications being infrequently outlined. Distinctly, monocystic manifestations correlated with an enhanced average survival duration [7]. Progressing to 2004, Hasegawa and colleagues chronicled control over lesions exceeding 70%, coupled with superior endocrinological outcomes amid predominantly cystic manifestations [6]. Furthermore, 2007 witnessed a report by Julow and associates, detailing an average decline in cystic volume by 9.6 milliliters among 60 individuals subjected to irradiation deploying yttrium-90, distinctively without the utilization of external beam radiotherapy (EBR) [36].

Within the context of the systematic review and meta-analyses conducted, intracystic irradiation maintains a safety profile in alignment with historical precedents. Concurrently, an inclination towards efficacious outcomes emerges concerning disease mastery, recurrence mitigation, and the minimization of visual and endocrine ramifications, notably under particular circumstances. In an investigation by Hu and colleagues encompassing 32 subjects with recurrent CPs, an exploration into phosphorus brachytherapy’s efficacy was undertaken. The findings discerned that brachytherapy-responsive lesions shared distinct attributes, including cystic walls measuring less than 2 millimeters, uniformity and low-density in cystic fluid within multicystic configurations, displaying subtle, pale, or deep yellow hues, and a diminished prevalence of vascular endothelial growth factor receptor 2 (VEGFR-2). In contrast, lesions demonstrating radio-resistance exhibited heightened VEGFR-2 expression, cystic walls exceeding 2 millimeters, heterogeneous cyst densities in multicystic structures with a brownish or soy sauce-like chroma in cystic fluid, and a preponderance of solid elements [23]. Adding to the discourse, Albright and associates, in their publication, elucidated the outcomes of a personalized approach to craniopharyngioma, involving a cohort of 12 pediatric subjects. Application of P-32 brachytherapy facilitated cystic reduction in 10 out of 12 instances. Nonetheless, a mean surveillance duration of 25 months precludes definitive assertions regarding the protracted prognosis of these pediatric cases [5]. Extending the timeline, Zhao and team applied P-32 in intracavitary brachytherapy spanning 20 pediatric cases from 1981 to 2006. While the professed local neoplastic control rate stood at 100% within a 3–6 months post-therapeutic interval, supplemental interventions became a necessity for 14 subjects, attributable to the advancement of the irradiated cysts [37].

In our meta-analysis, the overall 1-year, 2–3 year, and 5-year progression free survival rates (PFS) are 75% (95%CI: 66-84%), 62% (95%CI: 52-72%) and 46% (95%CI: 36-56%), respectively. However, four studies included in the literature reviewed in this article received EBR and stereotactic radiosurgery prior to brachytherapy. It may affect the survival outcomes and toxicity of CPs patients. The subgroup analysis revealed that those who had received EBR or stereotactic radiosurgery prior to brachytherapy had a higher severity of visual and endocrine impairment compared to those who did not receive these treatments. Barriger et al. found that EBR was necessary to control craniopharyngiomas in some patients, it was primarily used to address problems related to progression of solid tumor components. It also found that delaying EBR as the more severe cranial nerve toxicities occurred in patients who had multiple recurrences and more aggressive interventions [27]. Pollock et al. reported there was a trend toward a greater number of patients developing visual deficits if they had prior EBRT, but this was not statistically significant [35]. Complications of treatment for craniopharyngiomas frequently include visual deterioration or pituitary gland dysfunction. Worsening of visual fields or visual acuity has been reported in 9–15% of patients after surgical resection, 0–34% after surgery followed by radiation, and 3.2–38% after Gamma Knife radiosurgery [27]. Compared to brachytherapy alone, EBR or stereotactic radiosurgery prior to brachytherapy for the treatment of CPs may improve efficacy, but it is also important to pay attention to the associated toxicity of this combined therapy.

Despite the comprehensive nature of meta-analyses, the present investigation acknowledges several pronounced limitations. Primarily, the inclusion of a substantial quotient of retrospective studies within this meta-analysis potentially skews the integrative data, introducing an element of bias. The academic community recognizes the imperative for a greater proliferation of meticulously structured clinical trials alongside high-caliber prospective studies to corroborate these preliminary observations more robustly. Additionally, our meta-analysis only contained a few studies and the number of patients in each research was limited, some bias may exist in our study when the data were pooled.

## Conclusion

Conclusively, the outcomes delineated above advocate for the integration of brachytherapy as a good choice for managing CPs. The efficacy underscored in preceding observations posits this therapeutic approach as advantageous. However, there exists an unequivocal necessity for the initiation of additional randomized controlled trials. Such investigative endeavors would be instrumental in elucidating the specific patient demographics within the CP diagnosed population that would most significantly benefit from this treatment protocol.

### Electronic supplementary material

Below is the link to the electronic supplementary material.

Supplementary Material 1

Supplementary Material 2

## Data Availability

All data are available from the references provided.

## Abbreviations

CPs: Craniopharyngiomas
PRISMA: Preferred Reporting Items for Systematic Reviews and Meta-analyses guidelines
CI: Confidence interval
OS: Overall survival
PFS: Progression free survival rates
