# Supplementary material for: Brachytherapy in craniopharyngiomas: a systematic review and meta-analysis of long-term follow-up
Source: BMC Cancer. 2024 May 24;24:637. doi: 10.1186/s12885-024-12397-1 (PMC11127349; doi:10.1186/s12885-024-12397-1)
Supplement: Supplementary file 2 — Supplementary Material 2 [file 12885_2024_12397_MOESM2_ESM.docx]

Supplementary Table 1 Physical characteristics of β-radiation emitting radionuclides

| Radionuclide | Phosphorous-32 | Yttrium-90 |
| --- | --- | --- |
| Physical half-life (days) | 14.3 | 2.67 |
| Mean beta energy (MeV) | 0.69 | 0.93 |
| Half-value depth in soft tissue (mm) | 0.8 | 1.1 |

MeV mega-electron volts, mm millimetres.
